# Supplementary material for: HealthContradict: Evaluating biomedical knowledge conflicts in language models
Source: NPJ Digit Med. 2026 Jan 21;9:152. doi: 10.1038/s41746-025-02336-0 (PMC12901028; doi:10.1038/s41746-025-02336-0)
Supplement: Supplementary file 1 — Supplementary information [file 41746_2025_2336_MOESM1_ESM.pdf]

Supplementary Information

Evaluation Statistics

Statistic	Value
Instances	920
Questions	81
Documents	1,840
Max doc length (words)	30,444
Min doc length (words)	23
Avg doc length (words)	2,347
Earliest date	2012-02-10
Latest date	2019-04-26
Unique domains	1,403
Avg URL path length	6

Table S1. Statistics of HEALTHCONTRADICT dataset.

Domain	Count
`www.emfnews.org`	20
`www.drbriffa.com`	20
`www.healthline.com`	15
`cellphoneradiationtoday.com`	11
`www.quackometer.net`	10
`www.verywellhealth.com`	9
`www.medicalnewstoday.com`	9
`www.phantomvibrationsyndrome.com`	9
`www.webmd.com`	8
`www.livestrong.com`	8

Table S2. Top 10 most frequent domains in the HEALTHCONTRADICT dataset.

Another Case Study

Table S3 shows the case study of the same example in Figure **??** and Table **??**. Under the question – *"Can coffee help you lose weight?"* – both models are unsure of what to answer with their parametric knowledge. Moreover, MEDITRON3-8B made the wrong prediction `NO` with a probability score of 0.59. When adding the correct context, both models give a higher probability in the correct answer, `YES`. MEDITRON3-8B could change from an incorrect answer to a correct answer. However, when adding the incorrect context, both models boosted their probability in the incorrect answer `NO`. Both the finetuned biomedical model and its general domain counterpart fail to resist the incorrect context. When adding contradictory context, both models could identify the correct context and make the right prediction.

	Prompt Description	MEDITRON3-8B	LLAMA-3.1-8B-INSTRUCT
1	...**Question:** Can coffee help you lose weight?	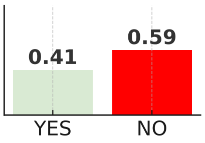	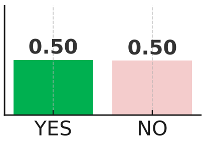
2	...**Context:** ... Green coffee can be taken before or after meals and it is also known to be useful for weight loss ...	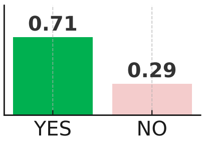	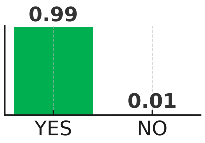
3	...**Context:** ... Does Coffee Can Magically Lose You Weight? Of Course Not! ...	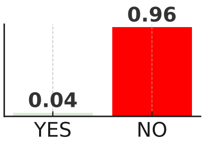	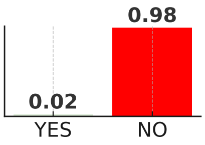
4	...**Context:** ... Green coffee can be taken before or after meals and it is also known to be useful for weight loss ...<br>... Does Coffee Can Magically Lose You Weight? Of Course Not! ...	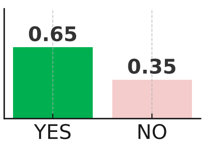	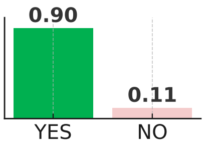
5	...**Context:** ... Does Coffee Can Magically Lose You Weight? Of Course Not! ...<br>... Green coffee can be taken before or after meals and it is also known to be useful for weight loss ...	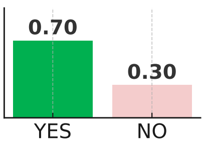	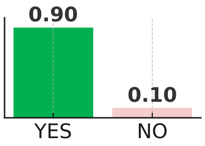

Table S3. Model probability Scores for MEDITRON3-8B and LLAMA-3.1-8B-INSTRUCT on the Question "Can coffee help you lose weight?"

Disease and Condition Categories of Health Questions in HEALTHCONTRADICT

Table S4. Health Questions and Their Associated Disease or Condition Categories

Disease/Condition	Question	TREC ID
AIDS	Can HIV be transmitted through sweat?	183
AIDS	Did AIDS come from chimps?	181
AIDS	Is male circumcision helpful in reducing heterosexual men's chances of getting HIV?	12
Alcohol use disorder	Can benzos (benzodiazepines) help with alcohol withdrawal?	41
Anxiety disorders	Can l-theanine supplements reduce stress and anxiety?	131
Arthritis	Can you use WD-40 for arthritis?	155
Arthritis	Do magnetic wrist straps help with arthritis?	164
Arthritis	Can copper bracelets reduce the pain of arthritis?	139
Asthma	Can vitamin D supplements improve the management of asthma?	146
Asthma	Does yoga improve the management of asthma?	107
Athlete's foot	Can fungal creams treat athlete's foot?	140
Atopic dermatitis	Can dupixent treat eczema?	118
Atopic dermatitis	Are probiotics an effective treatment for eczema?	42
Autism	Are vaccines linked to autism?	158
Burns	Should I apply ice to a burn?	105

Disease/Condition	Question	TREC ID
Cancer	Does deli meat increase your risk of colon cancer?	190
Cancer	Can cell phones cause cancer?	154
Cancer	Can cancer be inherited?	157
Cancer	Can baking soda help to cure cancer?	159
Cancer	Does selenium help prevent cancer?	109
Cancer	Is amygdalin or laetrile an effective cancer treatment?	6
Cavities and tooth decay	Can oil pulling heal cavities?	192
Common cold	Does Vitamin C prevent colds?	187
Common cold	Does inhaling steam help treat common cold?	132
Common warts	Does duct tape work for wart removal?	104
Croup	Does steam from a shower help croup?	128
Dementia	Can folic acid help improve cognition and treat dementia?	103
Depression	Can music therapy help manage depression?	144
Diabetes	Can fruit juice increase the risk of diabetes?	163
Diabetes	Can cinnamon help people with diabetes?	40
Epilepsy	Can vitamins help manage epilepsy?	44
Erectile dysfunction	Do ACE inhibitors typically cause erectile dysfunction?	161
Eye problems in adults	Are carrots good for your eyes?	175
Fever	Is starving a fever effective?	108
Fever	Is a tepid sponge bath a good way to reduce fever in children?	102
Foreign object swallowed	Will drinking vinegar dissolve a stuck fish bone?	137
Hair loss	Can minoxidil treat hair loss?	129
Hemorrhoids	Does a high fiber diet help with hemorrhoids?	193
High cholesterol	Can exercise lower cholesterol?	178
High cholesterol	Can fish oil improve your cholesterol?	170
Hypertension	Can fermented milk help mitigate high blood pressure?	117
Iron deficiency anemia	Can eating dates help manage iron deficiency anemia?	136
Jet lag disorder	Can melatonin be used to reduce jet lag?	8
Keloid scar	Can applying vitamin E cream remove skin scars?	114
Kidney stones	Can crystals heal?	152
Knee Pain	Are squats bad for knees?	160
Low back pain in adults	Can exercises relieve lower back pain?	11
Low back pain in adults	Find documents that discuss using antidepressants for helping to manage or relieve lower back pain.	13
Low back pain in adults	Is lumbar traction an effective treatment for lower back pain?	38
Low back pain in adults	Can insoles treat back pain?	47
Migraine	Does Aleve relieve migraine headaches?	122
Migraine	Can the drug Imitrex (sumatriptan) treat acute migraine attacks?	120
Mild cognitive impairment	Can statins cause permanent cognitive impairment?	186
Mosquito bites	Can mosquito bites make you sick?	156
Muscle cramp	Can magnesium prevent muscle cramps?	16
Nausea and vomiting	Does ginger help with nausea?	199
Obesity	Is bariatric surgery effective for obesity?	50
Opioid Use Disorder	Is morphine addictive?	162
Osteoarthritis	Will at-home exercises manage hip osteoarthritis pain?	149
Osteoarthritis	Does Tylenol manage the symptoms of osteoarthritis?	143
Osteoarthritis	Can collagen supplements cure osteoarthritis?	153
Other	Are there health benefits to drinking your own urine?	167
Other	Is pink salt good for you?	168
Other	Is hydroquinone banned in Europe?	173
Other	Can chewing gum help lose weight?	179
Other	Do Himalayan salt lamps have health benefits?	184
Other	Can coffee help you lose weight?	188
Other	Does drinking lemon water help with belly fat?	189

Disease/Condition	Question	TREC ID
Other	Can grapefruit interfere with medication?	194
Other	Can vape pens be harmful?	195
Other	Is wifi harmful for health?	197
Ovarian cyst	Will taking birth control pills treat an ovarian cyst?	110
Pneumonia	Can antibiotics be use to treat community acquired pneumonia in children?	29
Post-extraction bleeding	Do tea bags help to clot blood in pulled teeth?	151
Pregnancy	Can a woman get pregnant while breastfeeding?	185
Pregnancy	Can an MRI harm my baby?	177
Pregnancy	Will taking zinc supplements improve pregnancy?	111
Rheumatoid arthritis	Is sulfasalazine an effective treatment for rheumatoid arthritis?	49
Spinal cord injury	Can steroids be used as a treatment for spinal cord injury?	20
Tick bites	Can I remove a tick by covering it with Vaseline?	134
Whooping cough	Can antibiotics be used as a treatment for whooping cough (pertussis)?	28
